# Species affiliation of the complete mitochondrial genome of Daurian ground squirrel *Spermophilus dauricus* Brandt, 1843 (Rodentia, Sciuridae)

**DOI:** 10.1080/23802359.2017.1331324

**Published:** 2017-05-24

**Authors:** Svetlana Kapustina, Oleg Brandler

**Affiliations:** Laboratory of Genome Evolution and Speciation, Koltzov Institute of Developmental Biology of Russian Academy of Sciences, Vavilova 26, Moscow, Russia

**Keywords:** Mitochondrial genome, KR534854, *Sermophilus dauricus*, *Sciurus vulgaris*, Xerinae

## Abstract

The complete mitochondrial genome of Daurian ground squirrel (*Spermophilus dauricus*) (Genbank NCBI KR534854) was tested by comparison with mtDNA markers isolated from *Sciurus vulgaris* mitochondrial genome and *S. dauricus* samples with reliable species diagnose. A high similarity between KR534854 and *S. vulgaris* was found but not with *S. dauricus*. It seems that the mitochondrion (KR534854) belongs not to *S. dauricus* but to one of the species of the genus *Sciurus*. Monophylies of Xerinae and Sciurinae were tested by using mtDNA control region. The monophyly of Xerinae is supported by mtDNA data provided the KR534854 sequence is excluded from an analysis.

The first complete mitochondrial genome of Daurian ground squirrel (*Spermophilus dauricus* Brandt, 1843) (GenBank NCBI KR534854) was published in ‘Mitochondrial DNA Part A’ volume 27, no. 4 (Jin et al. 2016). There are no data about the place of capture of the examined individual in this publication. Authors studied phylogenetic relationships of *S. dauricus* and some different genera of Sciuridae using their complete mitochondrions. Based on this analysis, authors concluded ‘*Spermophilus dauricus* formed the sister group to the Pteromyini tribe’ that is unexpected.

We studied a variability of mtDNA control region (CR) complete sequences of 64 *S. dauricus* from 20 locations in Russia and Mongolia, including a region of its terra typica (Torei-Noor Lake, Transbaikalia). Samples are kept in the collection of frozen and fixed tissues of wild animals at Koltzov Institute of Developmental Biology of Russian Academy of Sciences.

We have tried to use in our analysis a CR extracted from the *S. dauricus* mitochondrial genome (KR534854) to evaluate differences between subspecies *S. d. dauricus* and Chinese Daurian ground squirrels. The analysis shows that the difference between our data and the KR534854 CR is higher than within the genus *Spermophilus* (well over than the differences between well-diverged species *S. pallidicauda* and *S. dauricus*).

We tested the KR534854 sequence using BLAST NCBI program and found a high similarity between this sequence and Red squirrel (*Sciurus vulgaris*) mtDNA according to three mtDNA markers (*Cyt-b*, 97%; *COI*, 96%; *CR*, 98%). We further tested the KR534854 mitochondrion using the same mtDNA markers isolated from *S. dauricus* samples with reliable species diagnosis (Harrison et al. 2003; Ermakov et al. 2015). Higher differences were observed between the mitochondrion KR534854 and *S. dauricus* in comparison with the previous: *Cyt-b* (AF157899), 82%; *COI* (KM537955), 86%; *CR* (KY611535), 83%. Genetic distance between *Sciurus vulgaris* (AJ238588) and KR534854 (d_p_= 0.005) is subspecies level. We conclude that the mitochondrion (KR534854) belongs not to *S. dauricus* but to one species of the genus *Sciurus*, probably *S. vulgaris*.

It is necessary to note that using this mitochondrion in research and expertise as *S. dauricus* may lead to significant mistakes. For example, Zhang et al. (2016) studied phylogenetic relationships in family Sciuridae using 13 protein-coding genes from 20 species mitochondrions. They detected high similarity between ‘*S. dauricus*’ (KR534854) and *S. vulgaris* (AJ238588) and concluded that ‘*Spermophilus dauricus* (Xerinae) within subfamily Sciurinae is a sister clade to *Sciurus vulgaris* (Sciurinae). The monophyly of Xerinae is failed to support in this study.’ This conclusion is mistaken in view of the error in species identification of the KR534854 mitochondrion.

We constructed ML phylogenetic tree (Figure 1) using mtDNA CRs extracted from some mitochondrions studied by Zhang et al. (2016) and our CR sequences of *S. dauricus* KY611535, KY611491 to test monophyly of Xerinae and Sciurinae.

**Figure 1.**
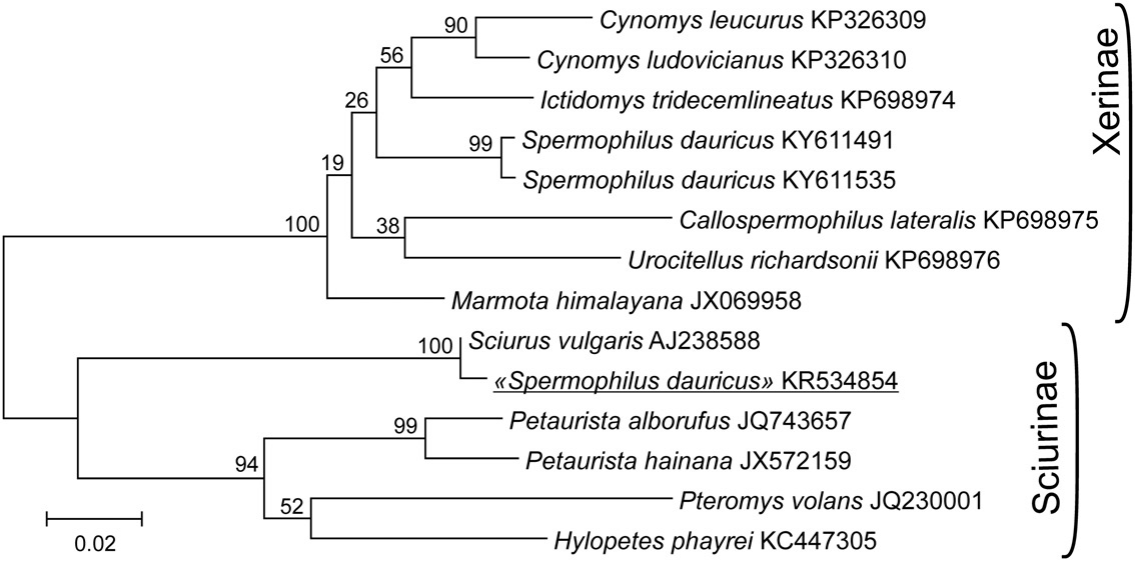
Maximum-likelihood phylogenetic tree of the relationships among 12 species of Marmotini based on mtDNA control region sequences is constructed in MEGA6. Numbers above branches specify bootstrap percentages (1000 replications). The GenBank numbers for the mitochondrial genomes of all species are also shown.

Our analysis shows that these sequences form two clades corresponding to subfamilies Sciurinae and Xerinae. Taxa are distributed among subfamily clades according to their traditional classification. Only a CR of the ‘*S. dauricus’* KR534854 clustered together with *S. vulgaris*. Therefore, a monophyly of Xerinae is supported by mtDNA data provided that KR534854 sequence is not included in the analysis.
